# *N*,*N*′-[1,4-Phenyl­enebis(imino­carbon­yl)]bis­(l-phenyl­alanine) tetra­hydro­furan disolvate

**DOI:** 10.1107/S2414314623007435

**Published:** 2023-09-08

**Authors:** Manuel Stapf, Anke Schwarzer

**Affiliations:** a Institut für Organische Chemie, Technische Universität Bergakademie Freiberg, Leipziger Strasse 29, 09599 Freiberg, Germany; Benemérita Universidad Autónoma de Puebla, México

**Keywords:** crystal structure, urea, amino acid, hydrogen bonding, tetra­hydro­furan solvate

## Abstract

The title compound, C_26_H_26_N_4_O_6_·2C_4_H_8_O, representing a bis-urea with terminal phenyl­alanine units, crystallized with two tetra­hydro­furan (THF) mol­ecules. The main mol­ecule is located on a crystallographic twofold axis, while the solvent mol­ecule is disordered over two positions, with occupancies of 0.571 (15) and 0.429 (15). The host mol­ecules are linked by N—H⋯O=C hydrogen bonds and C—H⋯O contacts with *R*_2_^1^(6) and *R*_2_^1^(7) ring motifs. The THF mol­ecules enclosed in the crystal are connected to the bis-urea compound *via* O—H⋯O and C—H⋯O inter­actions.

## Abstract 2

In the crystal structure of the title bis-urea derivative, the host mol­ecules are linked by N—H⋯O=C hydrogen bonds and C—H⋯O contacts with 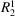 (6) and 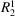 (7) ring motifs.

## Graphical abstract

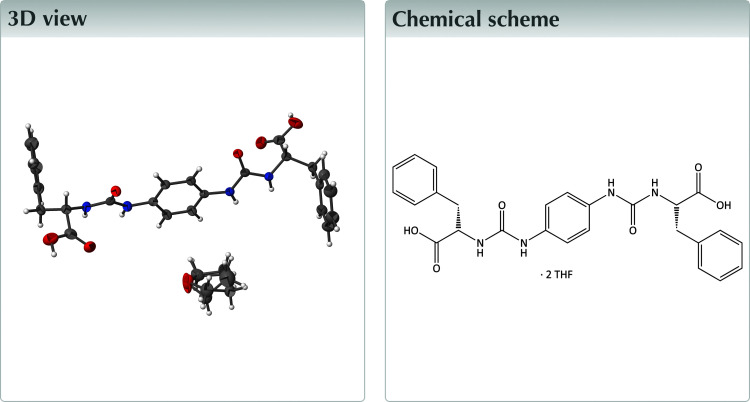

## Structure description

Bis-urea compounds containing a central 1,4-phenyl­ene unit have been shown to be suitable mol­ecules for anion recognition (Stapf *et al.*, 2015 ▸; Casula *et al.*, 2016 ▸; Manna *et al.*, 2018 ▸; Manna & Das, 2019 ▸, 2020 ▸; Das *et al.*, 2020 ▸). In this context, we introduced compounds combining this scaffold and various amino acids [such as l-valine, l-leucine, l-proline, (*R*)-3-piperidine­carb­oxy­lic acid, l-threonine or even l-phenyl­alanine], whose amino group is part of the urea moiety, among them the title compound, possessing l-phenyl­alanine units (Stapf *et al.*, 2015 ▸). Furthermore, we have already reported the crystal structure of a supra­molecular coordination polymer of the title compound with lead(II) (Stapf *et al.*, 2012 ▸). In the present article, we describe the crystal structure of the tetra­hydro­furan (THF) disolvate.

**Figure 1:**
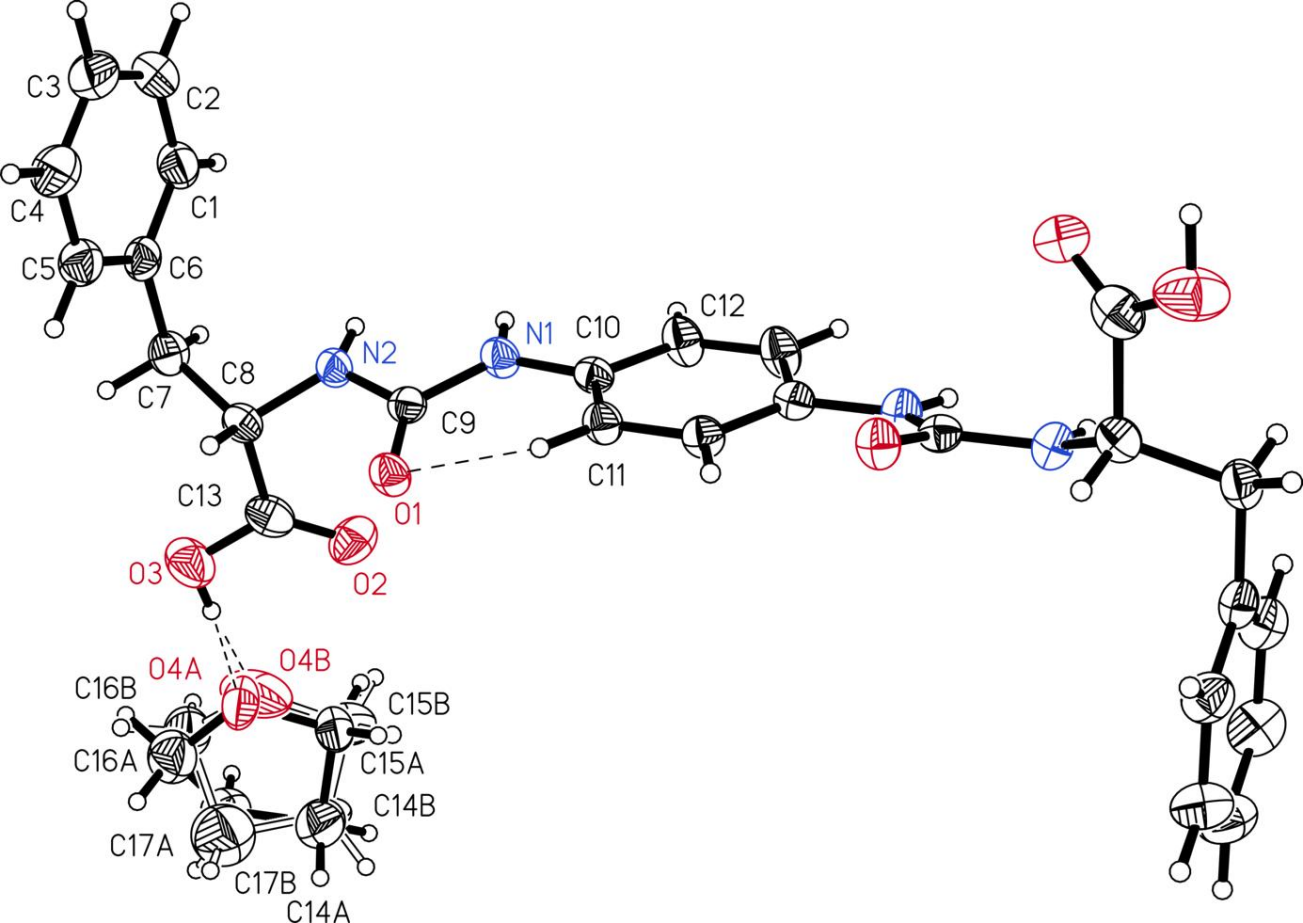
The mol­ecular structure of the title compound, including the atom-numbering scheme. Atoms are drawn with displacement ellipsoids at the 50% probability level. The intra­molecular C—H⋯O inter­action, as well as the inter­molecular hydrogen bonding between the carb­oxy group and the THF mol­ecule, are shown as dashed lines. Both disordered parts (57:43) of the THF mol­ecule are displayed. Unlabelled atoms are generated by the symmetry operation −*x* + 1, −*y*, *z*.

The title compound was found to crystallize in the tetra­gonal space group *I*4_1_ with half a mol­ecule of the bis-urea compound and one THF mol­ecule (Fig. 1 ▸), which is disordered over two positions (57:43). Within a single mol­ecule possessing a twofold rotation axis, the plane of the phenyl­ene unit includes a dihedral angle with the peripheral arene rings of 88.4 (1)° and with the planes of the urea moieties of 19.4 (2)°. This small angle may be associated with an intra­molecular C—H⋯O inter­action (H⋯O = 2.35 Å) between the phenyl­ene core and the urea moiety. Furthermore, the carb­oxy group is almost perpendicular to the central aromatic ring, showing a dihedral angle of 82.9 (1)°, and the phenyl­ene units of adjacent mol­ecules are oriented orthogonal with respect to each other.

**Figure 2:**
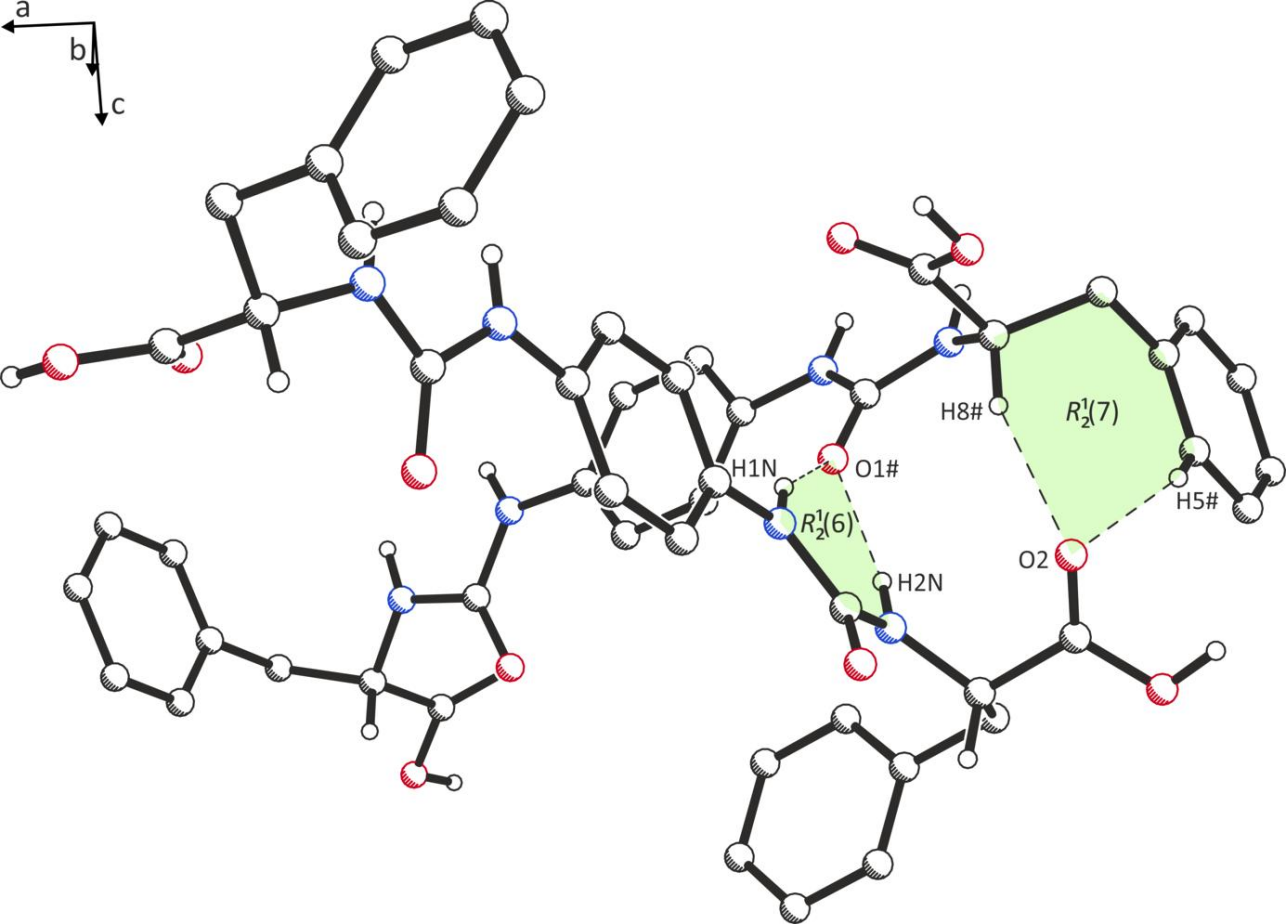
Excerpt of the crystal packing showing the 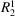 (6) and 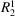 (7) ring motifs of the N—H⋯O=C and C—H⋯O=C(OH) inter­actions drawn as dashed lines. The THF mol­ecules and the H atoms not involved in the inter­actions have been omitted for clarity.

The dominant inter­molecular inter­actions between urea moieties of neighbouring mol­ecules are inverse bifurcated hydrogen bonds of the N—H⋯O=C type [H⋯O = 2.08 (4) and 2.32 (3) Å; Table 1 ▸], which can be described by the graph set 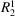 (6) (Etter, 1990 ▸; Fig. 2 ▸). Unlike in the previously published coordination polymer (Stapf *et al.*, 2012 ▸), in which the urea groups form two-dimensional hydrogen-bridged ribbons (H⋯O = 2.06–2.26 Å), the structure presented here is characterized by supra­molecular chains [graph set *C*(4)]. The angle between the planes of adjacent urea moieties is 83.7 (1)°, thus they are nearly perpendicular to one other. Such a motif is also well known in the literature (for examples, see: Albrecht *et al.*, 2002 ▸; Berkessel *et al.*, 2006 ▸; Saxena *et al.*, 2014 ▸; Shugrue *et al.*, 2019 ▸). The N atoms do not act as acceptors for hydrogen bonds. Instead, the linkage of two adjacent mol­ecules is supported by the formation of C—H⋯O=C contacts (H⋯O = 2.55 and 2.70 Å) between the C—H groups of phenyl­alanine and an O atom of a carb­oxy group which acts as a bifurcated acceptor [graph set 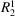 (7); Fig. 2 ▸].

The crystal structure exhibits cavities which are occupied by THF mol­ecules requiring about 961 Å^3^ (corresponding to about 29% of the unit-cell volume). The cavities are bounded by the nonpolar phenyl­ene and arene units of the title compound. In addition, the carb­oxy groups point into the inter­ior of these cavities and form O—H⋯O hydrogen bonds with the THF O atom [H⋯O = 1.73 (3) Å]. Further stabilization of the mol­ecular network, each involving the THF mol­ecules, is realized by C—H⋯O contacts with the carb­oxy group of an adjacent mol­ecule (H⋯O = 2.78 Å) and weak C—H⋯π contacts (H⋯*Cg* = 2.61–3.00 Å) with the central benzene core or peripheral arene substituents.

## Synthesis and crystallization

The synthetic and spectroscopic details for the title compound have been reported previously (Stapf *et al.*, 2012 ▸, 2015 ▸). Single crystals suitable for X-ray analysis were obtained as colourless prisms by slow evaporation of a saturated solution of the bis-urea compound in tetra­hydro­furan.

## Refinement

Crystal data, data collection and structure refinement details are summarized in Table 2 ▸. The H atoms at N1 and N2 were located in a difference Fourier map and refined freely. The H atom at O3 was also located in the difference Fourier map but refined using a DFIX restraint at 0.84 (2) Å. Other H atoms were included using a riding model starting from calculated positions (aromatic C—H = 0.95 Å, methyl­ene C—H = 0.99 Å, and alkyl C—H = 1.00 Å). The *U*_iso_(H) values were fixed at 1.2 times the equivalent *U*_eq_ value of the parent C atoms. The THF solvent mol­ecule is disordered over at least two positions [refined occupancies 0.571 (15) and 0.429 (15)]. Therefore, the solvent mol­ecule was refined using ISOR for C16*A*, C16*B*, C17*A* and C17*B* (approximate isotropic behaviour) and SADI (same distances over pairs of bonded atoms) restraints (Sheldrick, 2015*b* ▸). The absolute structure of the title compound has been assigned by reference to an unchanging chiral centre in the synthetic procedure, not by anomalous dispersion effects in the diffraction experiment.

## Supplementary Material

Crystal structure: contains datablock(s) I. DOI: 10.1107/S2414314623007435/bh4077sup1.cif

Structure factors: contains datablock(s) I. DOI: 10.1107/S2414314623007435/bh4077Isup2.hkl

Supporting information file. DOI: 10.1107/S2414314623007435/bh4077Isup3.cml

CCDC reference: 2290568

Additional supporting information:  crystallographic information; 3D view; checkCIF report

## Figures and Tables

**Table 1:** Hydrogen-bond geometry (Å, °) *Cg* is defined as the centre of gravity of the rings: *Cg*1 is ring C1–C6 and *Cg*2 is C10/C11/C12/C10′/C11′/C12′, with primed atoms generated by the symmetry code (−*x* + 1, −*y*, *z*).

*D*—H⋯*A*	*D*—H	H⋯*A*	*D*⋯*A*	*D*—H⋯*A*
N1—H1*N*⋯O1^i^	0.84 (4)	2.32 (3)	3.091 (3)	153 (3)
N2—H2*N*⋯O1^i^	0.88 (3)	2.08 (4)	2.937 (3)	163 (3)
O3—H3*O*⋯O4*A* ^ii^	0.90 (3)	1.73 (3)	2.623 (7)	175 (7)
C5—H5⋯O2^iii^	0.95	2.55	3.464 (4)	161
C8—H8⋯O2^iii^	1.00	2.70	3.629 (4)	155
C11—H11⋯O1	0.95	2.35	2.916 (3)	118
C14*A*—H14*B*⋯O3^iv^	0.99	2.78	3.581 (18)	139
C16*A*—H16*A*⋯*Cg*1^v^	0.99	2.61	3.497 (8)	149
C16*B*—H16*D*⋯*Cg*1^v^	0.99	2.82	3.449 (10)	122
C17*A*—H17*B*⋯*Cg*2^vi^	0.99	3.00	3.574 (8)	118

Symmetry codes: (i) 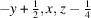 ; (ii) 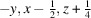 ; (iii) 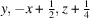 ; (iv) 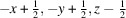 ; (v) 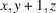 ; (vi) 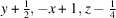 .

**Table 2:** Experimental details

Crystal data	
Chemical formula	C_26_H_26_N_4_O_6_·2C_4_H_8_O
*M*_r_	634.71
Crystal system, space group	Tetragonal, *I*4_1_
Temperature (K)	153
*a*, *c* (Å)	13.632 (4), 17.507 (6)
*V* (Å^3^)	3253 (2)
*Z*	4
Radiation type	Mo *K*α
μ (mm^−1^)	0.09
Crystal size (mm)	0.10 × 0.05 × 0.04

Data collection	
Diffractometer	Stoe IPDS 2T
No. of measured, independent and observed [*I* > 2σ(*I*)] reflections	25670, 3554, 3269
*R*_int_	0.028
(sin θ/λ)_max_ (Å^−1^)	0.639

Refinement	
*R*[*F* ^2^ > 2σ(*F* ^2^)], *wR*(*F* ^2^), *S*	0.040, 0.107, 1.05
No. of reflections	3554
No. of parameters	266
No. of restraints	95
H-atom treatment	H atoms treated by a mixture of independent and constrained refinement
Δρ_max_, Δρ_min_ (e Å^−3^)	0.31, −0.20

Computer programs: *X-AREA* (Stoe & Cie, 2009 ▸), *X-RED* (Stoe & Cie, 2009 ▸), *SHELXT2018* (Sheldrick, 2015*a* ▸), *SHELXL2018* (Sheldrick, 2015*b* ▸), *XP* (Sheldrick, 2008 ▸), *WinGX* (Farrugia, 2012 ▸), *publCIF* (Westrip, 2010 ▸) and *shelXle* (Hübschle *et al.*, 2011 ▸).

## full crystallographic data

### Crystal data

**Table:**

C_26_H_26_N_4_O_6_·2C_4_H_8_O	*D*_x_ = 1.296 Mg m^−^^3^
*M**_r_* = 634.71	Mo *K*α radiation, λ = 0.71073 Å
Tetragonal, *I*4_1_	Cell parameters from 1266 reflections
*a* = 13.632 (4) Å	θ = 3.1–26.7°
*c* = 17.507 (6) Å	µ = 0.09 mm^−^^1^
*V* = 3253 (2) Å^3^	*T* = 153 K
*Z* = 4	Chunk, colourless
*F*(000) = 1352	0.10 × 0.05 × 0.04 mm

### Data collection

**Table:**

STOE IPDS 2T diffractometer	3269 reflections with *I* > 2σ(*I*)
Radiation source: sealed X-ray tube, 12 x 0.4 mm long-fine focus	*R*_int_ = 0.028
Plane graphite monochromator	θ_max_ = 27.0°, θ_min_ = 3.0°
Detector resolution: 6.67 pixels mm^-1^	*h* = −17→17
rotation method scans	*k* = −17→17
25670 measured reflections	*l* = −22→22
3554 independent reflections	

### Refinement

**Table:**

Refinement on *F*^2^	Primary atom site location: dual
Least-squares matrix: full	Secondary atom site location: difference Fourier map
*R*[*F*^2^ > 2σ(*F*^2^)] = 0.040	Hydrogen site location: mixed
*wR*(*F*^2^) = 0.107	H atoms treated by a mixture of independent and constrained refinement
*S* = 1.05	*w* = 1/[σ^2^(*F*_o_^2^) + (0.0557*P*)^2^ + 2.0532*P*] where *P* = (*F*_o_^2^ + 2*F*_c_^2^)/3
3554 reflections	(Δ/σ)_max_ = 0.002
266 parameters	Δρ_max_ = 0.31 e Å^−^^3^
95 restraints	Δρ_min_ = −0.20 e Å^−^^3^

### Fractional atomic coordinates and isotropic or equivalent isotropic displacement parameters (Å^2^)

**Table:**

	*x*	*y*	*z*	*U*_iso_*/*U*_eq_	Occ. (<1)
N1	0.38152 (16)	0.16933 (16)	0.54757 (12)	0.0285 (4)	
H1N	0.376 (2)	0.194 (2)	0.504 (2)	0.030 (8)*	
N2	0.27971 (16)	0.29428 (15)	0.58122 (12)	0.0278 (4)	
H2N	0.285 (2)	0.310 (2)	0.533 (2)	0.034 (8)*	
O1	0.31329 (14)	0.17368 (13)	0.66808 (10)	0.0302 (4)	
O2	0.11068 (16)	0.19008 (15)	0.57508 (14)	0.0445 (5)	
O3	0.02642 (16)	0.30373 (19)	0.63921 (15)	0.0529 (6)	
H3O	−0.023 (4)	0.263 (4)	0.627 (5)	0.14 (3)*	
C1	0.3225 (2)	0.5393 (2)	0.54681 (18)	0.0389 (6)	
H1	0.302627	0.524528	0.496096	0.047*	
C2	0.4061 (2)	0.5960 (2)	0.55894 (19)	0.0460 (7)	
H2A	0.442763	0.619510	0.516591	0.055*	
C3	0.4360 (2)	0.6185 (2)	0.6331 (2)	0.0497 (8)	
H3	0.493013	0.657049	0.641531	0.060*	
C4	0.3818 (3)	0.5840 (2)	0.69431 (19)	0.0466 (7)	
H4	0.401726	0.599088	0.744967	0.056*	
C5	0.2986 (2)	0.5276 (2)	0.68217 (16)	0.0380 (6)	
H5	0.262005	0.504595	0.724742	0.046*	
C6	0.26774 (19)	0.50397 (18)	0.60822 (16)	0.0313 (5)	
C7	0.17887 (19)	0.44008 (19)	0.59624 (15)	0.0327 (6)	
H7A	0.161232	0.440197	0.541378	0.039*	
H7B	0.122863	0.467660	0.625126	0.039*	
C8	0.19711 (18)	0.33349 (18)	0.62249 (14)	0.0283 (5)	
H8	0.213017	0.333795	0.678256	0.034*	
C9	0.32327 (17)	0.20934 (17)	0.60329 (13)	0.0252 (5)	
C10	0.43982 (17)	0.08384 (17)	0.55283 (14)	0.0250 (5)	
C11	0.47052 (18)	0.04159 (18)	0.62133 (14)	0.0275 (5)	
H11	0.450935	0.069967	0.668486	0.033*	
C12	0.4700 (2)	0.0413 (2)	0.48385 (14)	0.0341 (6)	
H12	0.449394	0.068988	0.436734	0.041*	
C13	0.1072 (2)	0.2663 (2)	0.60914 (15)	0.0370 (6)	
O4A	0.6924 (5)	0.1252 (4)	0.3577 (7)	0.048 (2)	0.571 (15)
C14A	0.5578 (8)	0.2254 (9)	0.3317 (12)	0.062 (5)	0.571 (15)
H14A	0.519420	0.224367	0.379670	0.074*	0.571 (15)
H14B	0.515206	0.247603	0.289262	0.074*	0.571 (15)
C15A	0.6031 (10)	0.1256 (10)	0.3148 (10)	0.046 (4)	0.571 (15)
H15A	0.559079	0.071968	0.331644	0.055*	0.571 (15)
H15B	0.616352	0.118003	0.259558	0.055*	0.571 (15)
C16A	0.7185 (6)	0.2206 (5)	0.3801 (6)	0.057 (2)	0.571 (15)
H16A	0.787187	0.234862	0.365513	0.068*	0.571 (15)
H16B	0.711966	0.228135	0.436102	0.068*	0.571 (15)
C17A	0.6488 (4)	0.2893 (4)	0.3391 (4)	0.0483 (19)	0.571 (15)
H17A	0.674677	0.308654	0.288402	0.058*	0.571 (15)
H17B	0.635496	0.348937	0.369627	0.058*	0.571 (15)
O4B	0.6865 (10)	0.1184 (7)	0.3439 (12)	0.081 (5)	0.429 (15)
C14B	0.5595 (10)	0.2278 (11)	0.3178 (12)	0.043 (4)	0.429 (15)
H14C	0.491571	0.225648	0.337787	0.052*	0.429 (15)
H14D	0.560401	0.268410	0.270882	0.052*	0.429 (15)
C15B	0.5969 (15)	0.1248 (10)	0.3012 (13)	0.046 (4)	0.429 (15)
H15C	0.549184	0.074772	0.318503	0.056*	0.429 (15)
H15D	0.609168	0.115831	0.245904	0.056*	0.429 (15)
C16B	0.7256 (7)	0.2178 (7)	0.3503 (8)	0.053 (3)	0.429 (15)
H16C	0.748952	0.243547	0.300663	0.064*	0.429 (15)
H16D	0.778608	0.222487	0.388781	0.064*	0.429 (15)
C17B	0.6294 (8)	0.2691 (10)	0.3774 (10)	0.088 (4)	0.429 (15)
H17C	0.610777	0.249281	0.429818	0.105*	0.429 (15)
H17D	0.634210	0.341453	0.374449	0.105*	0.429 (15)

### Atomic displacement parameters (Å^2^)

**Table:**

	*U*^11^	*U*^22^	*U*^33^	*U*^12^	*U*^13^	*U*^23^
N1	0.0345 (11)	0.0309 (10)	0.0202 (9)	0.0040 (9)	0.0040 (8)	0.0030 (8)
N2	0.0338 (11)	0.0261 (10)	0.0235 (10)	0.0025 (8)	0.0050 (8)	0.0009 (8)
O1	0.0355 (10)	0.0342 (9)	0.0209 (8)	0.0044 (7)	0.0033 (7)	0.0026 (7)
O2	0.0431 (11)	0.0363 (11)	0.0540 (13)	−0.0057 (8)	−0.0076 (10)	−0.0039 (9)
O3	0.0357 (11)	0.0657 (15)	0.0574 (14)	−0.0110 (10)	0.0096 (10)	−0.0175 (12)
C1	0.0424 (15)	0.0388 (14)	0.0355 (13)	0.0037 (12)	0.0062 (12)	−0.0026 (12)
C2	0.0465 (17)	0.0469 (17)	0.0445 (17)	−0.0021 (13)	0.0145 (14)	−0.0026 (13)
C3	0.0425 (16)	0.0437 (17)	0.063 (2)	−0.0089 (13)	0.0042 (15)	−0.0104 (15)
C4	0.0492 (18)	0.0451 (17)	0.0454 (16)	−0.0092 (14)	−0.0022 (13)	−0.0104 (13)
C5	0.0451 (16)	0.0353 (14)	0.0337 (14)	−0.0013 (12)	0.0016 (12)	−0.0016 (11)
C6	0.0337 (13)	0.0249 (11)	0.0353 (13)	0.0066 (10)	0.0010 (10)	−0.0003 (10)
C7	0.0294 (12)	0.0328 (13)	0.0360 (14)	0.0061 (10)	−0.0019 (10)	0.0012 (11)
C8	0.0277 (11)	0.0333 (12)	0.0240 (11)	0.0004 (10)	0.0008 (9)	0.0005 (9)
C9	0.0268 (11)	0.0263 (11)	0.0224 (11)	−0.0041 (9)	0.0001 (9)	−0.0019 (9)
C10	0.0250 (11)	0.0284 (11)	0.0218 (10)	−0.0018 (9)	0.0011 (9)	0.0006 (9)
C11	0.0309 (11)	0.0306 (12)	0.0210 (11)	0.0013 (10)	0.0007 (9)	−0.0018 (9)
C12	0.0396 (14)	0.0427 (15)	0.0199 (11)	0.0115 (12)	0.0000 (10)	0.0012 (10)
C13	0.0344 (13)	0.0479 (16)	0.0289 (13)	−0.0011 (12)	0.0001 (11)	0.0060 (11)
O4A	0.038 (3)	0.034 (3)	0.072 (5)	−0.003 (2)	−0.021 (3)	−0.001 (2)
C14A	0.048 (6)	0.054 (7)	0.083 (9)	0.003 (5)	−0.003 (5)	−0.012 (5)
C15A	0.037 (5)	0.059 (7)	0.043 (5)	−0.006 (4)	−0.002 (3)	−0.015 (4)
C16A	0.057 (3)	0.055 (3)	0.059 (3)	0.003 (2)	−0.009 (2)	−0.002 (2)
C17A	0.049 (3)	0.038 (2)	0.058 (3)	0.0014 (19)	0.006 (2)	0.000 (2)
O4B	0.113 (10)	0.064 (6)	0.065 (6)	0.058 (6)	−0.020 (5)	−0.008 (5)
C14B	0.040 (7)	0.047 (7)	0.042 (5)	0.024 (5)	−0.003 (4)	−0.003 (4)
C15B	0.056 (8)	0.025 (6)	0.058 (9)	0.009 (5)	0.011 (5)	0.010 (5)
C16B	0.052 (3)	0.053 (3)	0.055 (4)	0.001 (2)	−0.007 (3)	−0.003 (3)
C17B	0.088 (5)	0.087 (5)	0.089 (5)	0.009 (3)	−0.004 (3)	−0.004 (3)

### Geometric parameters (Å, º)

**Table:**

N1—C9	1.371 (3)	C11—H11	0.9500
N1—C10	1.413 (3)	C12—C12^i^	1.390 (5)
N1—H1N	0.84 (4)	C12—H12	0.9500
N2—C9	1.357 (3)	O4A—C16A	1.405 (8)
N2—C8	1.441 (3)	O4A—C15A	1.430 (8)
N2—H2N	0.88 (3)	C14A—C15A	1.522 (9)
O1—C9	1.241 (3)	C14A—C17A	1.522 (10)
O2—C13	1.199 (4)	C14A—H14A	0.9900
O3—C13	1.324 (4)	C14A—H14B	0.9900
O3—H3O	0.90 (3)	C15A—H15A	0.9900
C1—C2	1.393 (5)	C15A—H15B	0.9900
C1—C6	1.395 (4)	C16A—C17A	1.515 (8)
C1—H1	0.9500	C16A—H16A	0.9900
C2—C3	1.395 (5)	C16A—H16B	0.9900
C2—H2A	0.9500	C17A—H17A	0.9900
C3—C4	1.383 (5)	C17A—H17B	0.9900
C3—H3	0.9500	O4B—C15B	1.435 (10)
C4—C5	1.387 (4)	O4B—C16B	1.460 (10)
C4—H4	0.9500	C14B—C15B	1.521 (9)
C5—C6	1.399 (4)	C14B—C17B	1.521 (11)
C5—H5	0.9500	C14B—H14C	0.9900
C6—C7	1.507 (4)	C14B—H14D	0.9900
C7—C8	1.544 (3)	C15B—H15C	0.9900
C7—H7A	0.9900	C15B—H15D	0.9900
C7—H7B	0.9900	C16B—C17B	1.560 (10)
C8—C13	1.547 (4)	C16B—H16C	0.9900
C8—H8	1.0000	C16B—H16D	0.9900
C10—C11	1.395 (3)	C17B—H17C	0.9900
C10—C12	1.402 (3)	C17B—H17D	0.9900
C11—C11^i^	1.390 (5)		
			
C9—N1—C10	127.4 (2)	O3—C13—C8	111.8 (3)
C9—N1—H1N	116 (2)	C16A—O4A—C15A	111.0 (7)
C10—N1—H1N	116 (2)	C15A—C14A—C17A	101.4 (8)
C9—N2—C8	121.0 (2)	C15A—C14A—H14A	111.5
C9—N2—H2N	117 (2)	C17A—C14A—H14A	111.5
C8—N2—H2N	117 (2)	C15A—C14A—H14B	111.5
C13—O3—H3O	107 (5)	C17A—C14A—H14B	111.5
C2—C1—C6	120.8 (3)	H14A—C14A—H14B	109.3
C2—C1—H1	119.6	O4A—C15A—C14A	104.3 (7)
C6—C1—H1	119.6	O4A—C15A—H15A	110.9
C1—C2—C3	120.2 (3)	C14A—C15A—H15A	110.9
C1—C2—H2A	119.9	O4A—C15A—H15B	110.9
C3—C2—H2A	119.9	C14A—C15A—H15B	110.9
C4—C3—C2	119.4 (3)	H15A—C15A—H15B	108.9
C4—C3—H3	120.3	O4A—C16A—C17A	106.4 (6)
C2—C3—H3	120.3	O4A—C16A—H16A	110.5
C3—C4—C5	120.4 (3)	C17A—C16A—H16A	110.5
C3—C4—H4	119.8	O4A—C16A—H16B	110.5
C5—C4—H4	119.8	C17A—C16A—H16B	110.5
C4—C5—C6	121.0 (3)	H16A—C16A—H16B	108.6
C4—C5—H5	119.5	C16A—C17A—C14A	101.4 (6)
C6—C5—H5	119.5	C16A—C17A—H17A	111.5
C1—C6—C5	118.2 (3)	C14A—C17A—H17A	111.5
C1—C6—C7	121.5 (3)	C16A—C17A—H17B	111.5
C5—C6—C7	120.2 (2)	C14A—C17A—H17B	111.5
C6—C7—C8	111.9 (2)	H17A—C17A—H17B	109.3
C6—C7—H7A	109.2	C15B—O4B—C16B	107.1 (10)
C8—C7—H7A	109.2	C15B—C14B—C17B	105.2 (7)
C6—C7—H7B	109.2	C15B—C14B—H14C	110.7
C8—C7—H7B	109.2	C17B—C14B—H14C	110.7
H7A—C7—H7B	107.9	C15B—C14B—H14D	110.7
N2—C8—C7	109.0 (2)	C17B—C14B—H14D	110.7
N2—C8—C13	108.9 (2)	H14C—C14B—H14D	108.8
C7—C8—C13	112.6 (2)	O4B—C15B—C14B	104.0 (8)
N2—C8—H8	108.8	O4B—C15B—H15C	111.0
C7—C8—H8	108.8	C14B—C15B—H15C	111.0
C13—C8—H8	108.8	O4B—C15B—H15D	111.0
O1—C9—N2	123.1 (2)	C14B—C15B—H15D	111.0
O1—C9—N1	123.9 (2)	H15C—C15B—H15D	109.0
N2—C9—N1	113.0 (2)	O4B—C16B—C17B	97.6 (8)
C11—C10—C12	118.8 (2)	O4B—C16B—H16C	112.2
C11—C10—N1	124.4 (2)	C17B—C16B—H16C	112.2
C12—C10—N1	116.8 (2)	O4B—C16B—H16D	112.2
C11^i^—C11—C10	120.70 (14)	C17B—C16B—H16D	112.2
C11^i^—C11—H11	119.7	H16C—C16B—H16D	109.8
C10—C11—H11	119.7	C14B—C17B—C16B	98.8 (10)
C12^i^—C12—C10	120.52 (15)	C14B—C17B—H17C	112.0
C12^i^—C12—H12	119.7	C16B—C17B—H17C	112.0
C10—C12—H12	119.7	C14B—C17B—H17D	112.0
O2—C13—O3	124.4 (3)	C16B—C17B—H17D	112.0
O2—C13—C8	123.9 (3)	H17C—C17B—H17D	109.7
			
C6—C1—C2—C3	−0.1 (5)	C9—N1—C10—C12	161.9 (2)
C1—C2—C3—C4	−0.1 (5)	C12—C10—C11—C11^i^	−0.4 (4)
C2—C3—C4—C5	0.1 (5)	N1—C10—C11—C11^i^	−178.7 (3)
C3—C4—C5—C6	0.2 (5)	C11—C10—C12—C12^i^	−0.4 (5)
C2—C1—C6—C5	0.4 (4)	N1—C10—C12—C12^i^	178.1 (3)
C2—C1—C6—C7	−178.2 (3)	N2—C8—C13—O2	5.8 (4)
C4—C5—C6—C1	−0.5 (4)	C7—C8—C13—O2	126.8 (3)
C4—C5—C6—C7	178.2 (3)	N2—C8—C13—O3	−173.6 (2)
C1—C6—C7—C8	110.2 (3)	C7—C8—C13—O3	−52.6 (3)
C5—C6—C7—C8	−68.4 (3)	C16A—O4A—C15A—C14A	−16.5 (18)
C9—N2—C8—C7	167.2 (2)	C17A—C14A—C15A—O4A	34.2 (17)
C9—N2—C8—C13	−69.6 (3)	C15A—O4A—C16A—C17A	−8.6 (15)
C6—C7—C8—N2	−57.9 (3)	O4A—C16A—C17A—C14A	29.8 (13)
C6—C7—C8—C13	−178.9 (2)	C15A—C14A—C17A—C16A	−38.3 (15)
C8—N2—C9—O1	−18.6 (4)	C16B—O4B—C15B—C14B	−28 (2)
C8—N2—C9—N1	163.4 (2)	C17B—C14B—C15B—O4B	−6 (2)
C10—N1—C9—O1	−0.6 (4)	C15B—O4B—C16B—C17B	48.8 (18)
C10—N1—C9—N2	177.4 (2)	C15B—C14B—C17B—C16B	34 (2)
C9—N1—C10—C11	−19.7 (4)	O4B—C16B—C17B—C14B	−48.8 (16)

Symmetry code: (i) −*x*+1, −*y*, *z*.

### Hydrogen-bond geometry (Å, º)

*Cg* is defined as the centre of gravity of the rings: *Cg*1 is C1···C6; *Cg*2 is C10/C11/C12/C10'/C11'/C12' with primed atoms generated by symmetry -*x*+1, -*y*, *z*.

**Table:**

*D*—H···*A*	*D*—H	H···*A*	*D*···*A*	*D*—H···*A*
N1—H1*N*···O1^ii^	0.84 (4)	2.32 (3)	3.091 (3)	153 (3)
N2—H2*N*···O1^ii^	0.88 (3)	2.08 (4)	2.937 (3)	163 (3)
O3—H3*O*···O4*A*^iii^	0.90 (3)	1.73 (3)	2.623 (7)	175 (7)
C5—H5···O2^iv^	0.95	2.55	3.464 (4)	161
C8—H8···O2^iv^	1.00	2.70	3.629 (4)	155
C11—H11···O1	0.95	2.35	2.916 (3)	118
C14*A*—H14*B*···O3^v^	0.99	2.78	3.581 (18)	139
C16*A*—H16*A*···*Cg*1^vi^	0.99	2.61	3.497 (8)	149
C16*B*—H16*D*···*Cg*1^vi^	0.99	2.82	3.449 (10)	122
C17*A*—H17*B*···*Cg*2^vii^	0.99	3.00	3.574 (8)	118

Symmetry codes: (ii) −*y*+1/2, *x*, *z*−1/4; (iii) −*y*, *x*−1/2, *z*+1/4; (iv) *y*, −*x*+1/2, *z*+1/4; (v) −*x*+1/2, −*y*+1/2, *z*−1/2; (vi) *x*, *y*+1, *z*; (vii) *y*+1/2, −*x*+1, *z*−1/4.
